# Intracellular pH affects mitochondrial homeostasis in cultured human corneal endothelial cells prepared for cell injection therapy

**DOI:** 10.1038/s41598-022-10176-1

**Published:** 2022-04-15

**Authors:** Hideto Deguchi, Tomoko Yamashita, Nao Hiramoto, Yohei Otsuki, Atsushi Mukai, Morio Ueno, Chie Sotozono, Shigeru Kinoshita, Junji Hamuro

**Affiliations:** 1 Department of Ophthalmology, Kyoto Prefectural University of Medicine, 465 Kajii-cho, Hirokoji-agaru, Kawaramachi-dori, Kamigyo-ku, Kyoto, 602-0841 Japan; 2 Department of Frontier Medical Science and Technology for Ophthalmology, Kyoto Prefectural University of Medicine, Kyoto, Japan

**Keywords:** Diseases, Eye diseases, Corneal diseases

## Abstract

This study aimed to uncover the mechanism responsible for the clinical efficacy of cell injection therapy with fully differentiated cultured cells. Analysis of polarized expression of ion transporters on cultured human corneal endothelial cells (CECs) subpopulations (SPs) was performed. The intracellular pH (pHi) between two CEC SPs, distinct in the proportion of differentiated cells, was measured, and the association with mitochondrial respiration homeostasis was investigated. The effects of the ion transporter inhibition by their selective inhibitors or siRNA transfection were also explored. Na^+^/K^+^-ATPase, Aquaporin 1, SLC4A11, NBCe1, NHE1 as transporters, and ZO-1, were all selectively expressed in differentiated SPs, but were almost null in the cell-state-transitioned SPs. We also confirmed that the pHi of CEC SPs affected their mitochondrial respiration by modulating the expression of these ion transporters via inhibitors or siRNA transfection. Ion and water transporters might participate in the maintenance of pHi and mitochondria homeostasis in differentiated SPs, which may contribute, combined with integral barrier functions, to efficient water efflux. The differences in intracellular pH between the two SPs is attributed to variations in the expression profile of specific ion transporters and mitochondrial functions, which may associate with the efficacy of the SPs in cell injection therapy.

## Introduction

The current treatments for corneal endothelial failure are penetrating keratoplasty^1^, Descemet stripping automated endothelial keratoplasty^2, 3^, and Descemet membrane endothelial keratoplasty^4^. Although recent surgical developments have contributed to earlier visual recovery and lower complications, problems include the need for a donor cornea, cell loss due to surgical invasion, and graft failure^1–4^. We previously reported on our novel surgical procedure to inject cultured human corneal endothelial cells (CECs) into the anterior chamber Five-year postoperative results revealed that this CEC injection therapy has a safe, effective, and stable outcome (survived eyes, 10/11 with good best corrected visual acuity after 5 years)^5, 6^.

We previously reported that there is heterogeneity in CECs and that subpopulations (SPs) can be classified by analyzing their cell surface cluster of definition (CD) antigen^7, 8^; we succeeded in definitively distinguishing CEC SPs with high clinical efficacy from those with low efficacy^5, 6^. In particular, mitochondrial oxidative phosphorylation (OXPHOS) was dominant in the clinically efficacious CECs. In contrast, the non-efficacious CECs in a cell-state transition (CST)^9, 10^ and characterized de-differentiation, epithelial–mesenchymal transition (EMT), immaturity, and senescence^11, 12^ showed elevated glycolysis and OXPHOS. However, it is still unclear why specified differentiated CEC SPs with OXPHOS dominance would elicit a superior improvement of corneal transparency. One of the hypotheses is that the differentiated CEC SPs are more efficient in the water efflux from the corneal stroma to the anterior chamber through the corneal endothelium because of the cooperation of plural intracellular signaling pathways, but the underlying molecular mechanism has thus far remained elusive.

We have preliminarily reported that the proteome analysis of CEC SPs, revealing diverse ion transporters, including SLC4A11 and Na^+^/H^+^ exchanger (NHE) 1, was skewed for heterogeneous CEC SPs^12^. However, the physiological significance of this skewing among in situ CECs remains thus far unidentified.

Aquaporin 1 (AQP1) and SLC4A11 are critical players engaging in water efflux from the stroma to the anterior chamber through the corneal endothelium (CE)^13^. SLC4A11 has been shown to be mutated in late-onset Fuchs corneal dystroph**y** (FECD) and in congenital hereditary endothelial dystrophy (CHED) of CE^13–18^. Individuals with CHED have mutations in *SLC4A11*, which encodes a transmembrane protein in the SLC4 family of bicarbonate transporters.

Intracellular pH (pHi) is related to mitochondrial function and indirectly to cell differentiation in other cells^19–27^. NHE1 is a ubiquitous plasma membrane glycoprotein that plays a key role in pHi homeostasis^27^. The maintenance of glycolysis and OXPHOS-processing enzymes are highly dependent on pHi. Accordingly, pHi can modify the cellular metabolism critical in deciding the cell’s fate^20, 22, 23, 28, 29^.

Increased NHE1 activity has been considered important for cell differentiation to occur, at least in some cell types^16, 30^. A novel link between NHE1 and the regulation of EMT has also been discussed^31^. NHE1 facilitates cardiomyocyte development from embryonic stem (ES) cell^20^, and reportedly, Smad5 acts as a pHi sensor and maintains mitochondrial functions^24^. However, the role of NHE1 may vary among cell types^30^. In addition to NHEs, other regulators of pHi homeostasis have been clarified to link to diverse physiological activities^32^. Gao et al.^22^ reported that the pHi of mesenchymal stem cells (MSCs) is higher than that of normal differentiated cells.

In cultured bovine CECs, Li et al. suggested that intracellular bicarbonate ions lower pHi, which results in the elevation of the glycolytic system, and intracellular lactate gradient formation, which plays a role in efficient water efflux. This is the first report that pHi may be deeply associated with corneal transparency^33^. Note that the proliferative behavior of bovine CECs is distinct from that of human CECs.

In this context, we hypothesized that pHi in human CECs is associated with mitochondrial homeostasis and divergent among heterogeneous CEC SPs. In this study, we explored the polarized protein expression of ion transporters and water transporters in clinically effective differentiated CEC SPs. We also confirmed that the pHi of CEC SPs affected their mitochondrial respiration by modulating the expression of these ion transporters via inhibitors or siRNA transfection. Our results suggested that pHi regulated not only cell fate, such as differentiation versus CST, de-differentiation, or EMT, but also mitochondrial homeostasis, and was consequently associated with the efficient water efflux from the corneal stroma to the anterior chamber of the differentiated CEC SPs.

## Results

### Gene and protein expression of ion transporters

**Figure 1:**
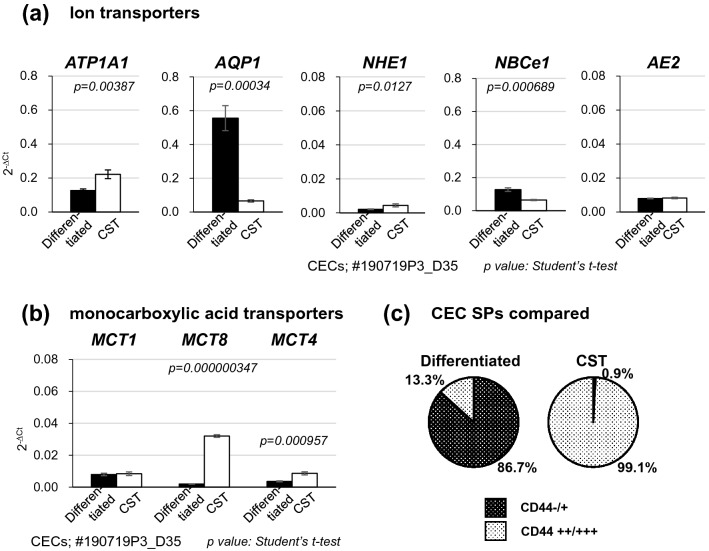
Gene and protein expression of ion and water transporters in differentiated and CST SPs. (**a**) Gene expression of ion and water transporters, *ATP1A1, AE2, AQP1, NHE1, and NBCe1.* The levels of *ATP1A1, AE2, AQP1, NBCe1 and NHE1* were normalized to the level of *GAPDH*. The results were presented as 2^−ΔCt^ (relative units of expression). (**b**) Gene expression of monocarboxylic acid transporters*, MCT1, MCT4*, and *MCT8*. The levels of *MCT1, MCT4,* and *MCT8* were normalized as above. The results were also presented as 2^−ΔCt^ (relative units of expression). (**c**) Cultured CECs were obtained from the same donor (two eyes, right and left mixed) and were incubated in the presence of 10-µM Y27632 (differentiated SPs, CD44-/+ proportion: 86.7%)) or 10-µM Y27632 + 1-µM SB431542 (SB4) + 10-µM SB203580 (SB2) + 5-ng/mL epidermal growth factor (EGF) (CST SPs, CD44-/+ proportion: 0.9%) from the third passage. The significance of the difference between the both types of CEC SPs was assessed by Student’s *t*-tests after confirmation by F tests. *P* < 0.05 was considered statistically significant.

In our previous proteome analysis, the expression level of the ion transporters was distinct between the differentiated SPs and the CST SPs^12^. However, this distinction was just a reflection of the distinct amounts of the corresponding proteins in the cell homogenates but not that in the intact cell entity of CEC SPs. Here, we performed RT-PCR (Fig. 1) to confirm the consistency of the gene activation and the total proteins extracted in the homogenates. Surprisingly, the gene expression of *ATP1A1*, the most abundantly expressed subtype of Na^+^/K^+^-ATPase in CECs, was found to be lower in differentiated SPs than in CST SPs (Fig. 1). Likewise, *NHE1*, one of the most pivotal regulators of pHi, was more highly expressed in the CST-SPs than in the differentiated SPs, in contrast to the inverse expression levels revealed by the previous proteome analysis^12^. In contrast, *NBCe1* was highly expressed in the CST SPs in the proteome analysis^12^ but was highly expressed in the differentiated CEC SPs in the RT-PCR. *AQP1* was highly expressed only in differentiated SPs (Fig. 1). The expression patterns in the RT-PCR were different from those observed in the proteome analysis, indicating the need to qualify the existence of these functional proteins not only by gene activation but also by immunofluorescence.

### Immunofluorescence detection of ion and water transporters

**Figure 2:**
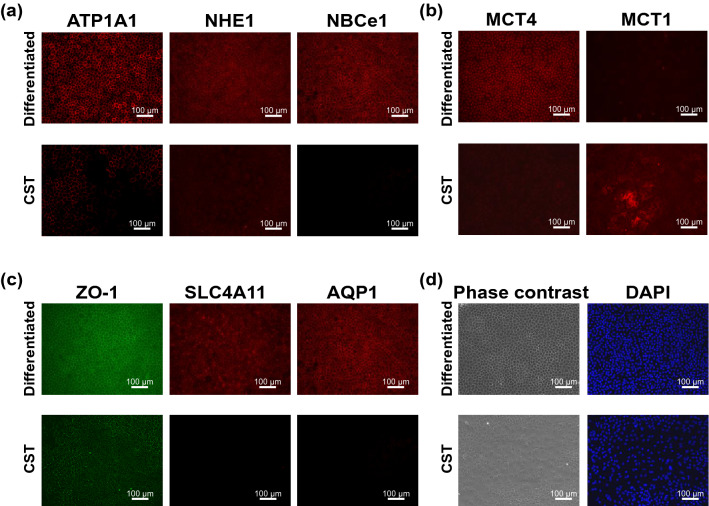
Immunofluorescence detection of ion transporters and water transporters in differentiated and CST- SPs. Na^+^/K^+^-ATP isoform ATP1A1, water transporter Aquaporin 1, SLC4A11, Bicarbonate transporter NBCe1, Na^+^-H^+^ exchanger NHE1 and ZO-1 as an endothelial barrier to complements fluid transport, were all selectively expressed in differentiated SPs, but were almost null in the cell-state-transitioned SPs. Monocarboxylic acid transporter 4 (MCT4) was expressed in the former, but not in the latter SPs, whereas the isomer MCT1 was selectively expressed in the latter SPs, but not in the former SPs. Differentiated SPs were obtained from the same donor (two eyes, right and left mixed) and were incubated in the presence of 10-µM Y27632 (differentiated SPs, CD44-/ + proportion: 86.7%). CST-SPs were obtained from the separate donor (two eyes, right and left mixed) and were incubated in the presence of 10-µM Y27632 + 1-µM SB431542 (SB4) + 10-µM SB203580 (SB2) + 5-ng/mL epidermal growth factor (EGF). The differentiated SPs were fixed at 37 days of the second passage, and the CST SPs were fixed at 38 days of the second passage. Bars: 100 µm. Experiments were repeated four times.

The immunofluorescence analysis was depicted in Fig. 2 divided to four functional groups; (a) Ion transporters, (b) Monocarboxylic acid transporters, (c) Transporters of water and (d) cell morphology. The immunofluorescence analysis showed that Na^+^/K^+^-ATPase, ZO-1, NHE1, NBCe1, SLC4A11, AQP1 and MCT4 were highly expressed selectively in differentiated SPs, while MCT1 was highly expressed in CST SPs (Fig. 2). Although not uniform, RT-PCR, proteome analysis and immunofluorescence analysis showed distinct expression levels of ion and water transporters between two SPs. Ion transporters, Na^+^/K^+^-ATPase, NHE1 and NBCe1, playing a role in regulating intracellular pH, were all highly expressed only in differentiated CECs (Fig. 2a) With respect to the water efflux from the corneal stroma to the anterior chamber through the endothelium, SLC4A11 and AQP1 performed critical roles, as well as ZO-1^13^. These two proteins and ZO-1 showed polarized expression only in differentiated SPs, while in CST SPs, the expressions of both SLC4A11 and AQP1, as well as that of ZO-1, were almost null (Fig. 2c). The expression profile of these proteins was also repeatedly confirmed by immunofluorescence with the CECs produced in the cell processing center (CPC) for the clinical setting (data not shown).

### pHi difference between differentiated and CST CEC SPs

**Figure 3:**
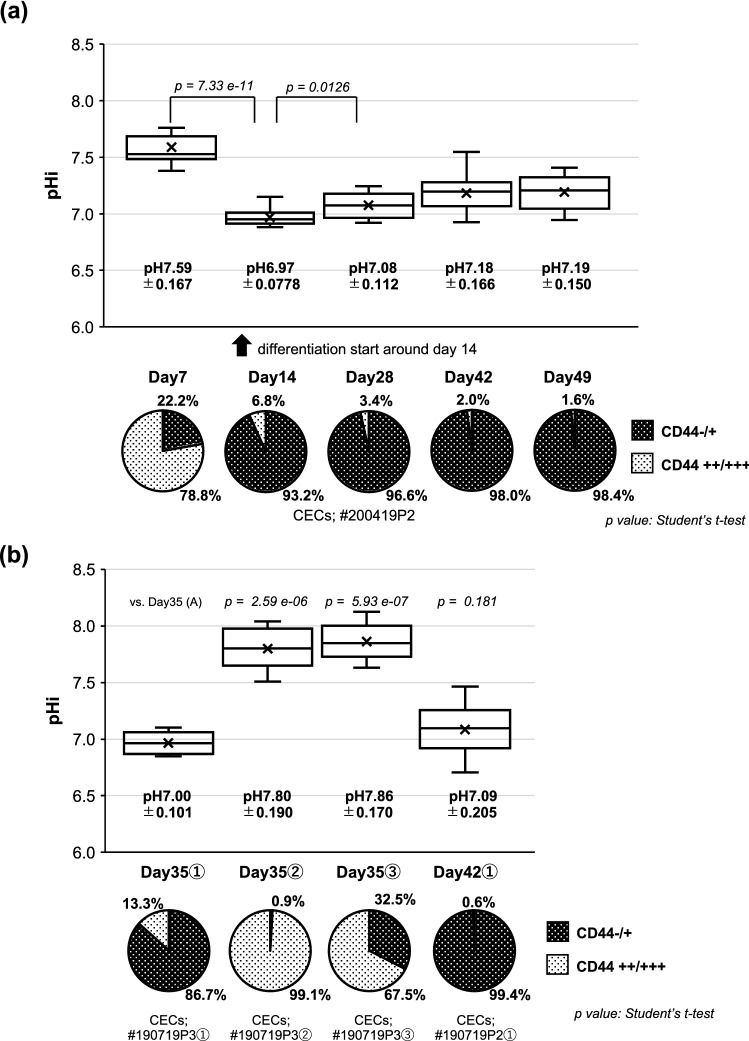
The alteration of pHi along line with differentiation in culture and the pHi difference between differentiated and CST-SPs. The pie graphs indicate the proportion of two CEC SPs, namely differentiated- and the CST-CEC SPs. (**a**) The alteration of pHi during the culture period was monitored on day 7, 14, 28, 42, and 49 (above), and the percentages of CD 44 -/+ cells and CD 44 ++/+++ cells (CST cells) were obtained from flow cytometry analysis (below). The increase of the proportion of CD44-/+ SPs means that the differentiation was started already around day 14. Cultured CECs were obtained from the same donor and were incubated in the presence of 10-µM Y27632. (**b**) Variation of pHi with different proportions of CD44-/+ phenotypes. Each SP was obtained from the same donor and incubated with Y27632 (①), plus SB4, SB2, and EGF (②), or plus SB4 and EGF (③). For (**a**) and (**b**); ( ×) marks indicate the average of pHi (n = 12). The significance of the difference was assessed by Student’s *t*-tests after confirmation by F tests. *P* < 0.05 was considered statistically significant.

On the basis of the distinctive expression of ion transporters among the SPs described and the previous findings of divergent energetic requirements among CEC SPs, it can be easily hypothesized that pHi might be variable among differentiated SPs and CST SPs. Considering the stepwise alteration of CECs, proliferation, differentiation, and maturation during culture, we monitored the alteration of pHi during the culture period and found that pHi, higher on day 7 after cell seeding, corresponding to the proliferative stage before the differentiation of CECs, dropped significantly on day 14 when CEC differentiation was initiated and then gradually increased until it maintained almost the same levels between day 42 and day 49 (Fig. 3a). The kinetics corresponded closely to the alteration of the increased ratios of the differentiated CEC SPs and the mitochondrial OXPHOS that we reported recently^10^. The pHi of differentiated SPs expressing the stable CD44 -/+  phenotypes on day 35 or day 42 was almost in the neutral range and was lower than that of CD44 ++/+++ CST SPs on day 35 (Fig. 3b), consistent with our preliminary data^12^.

### pHi regulation by culture environmental factors

**Figure 4:**
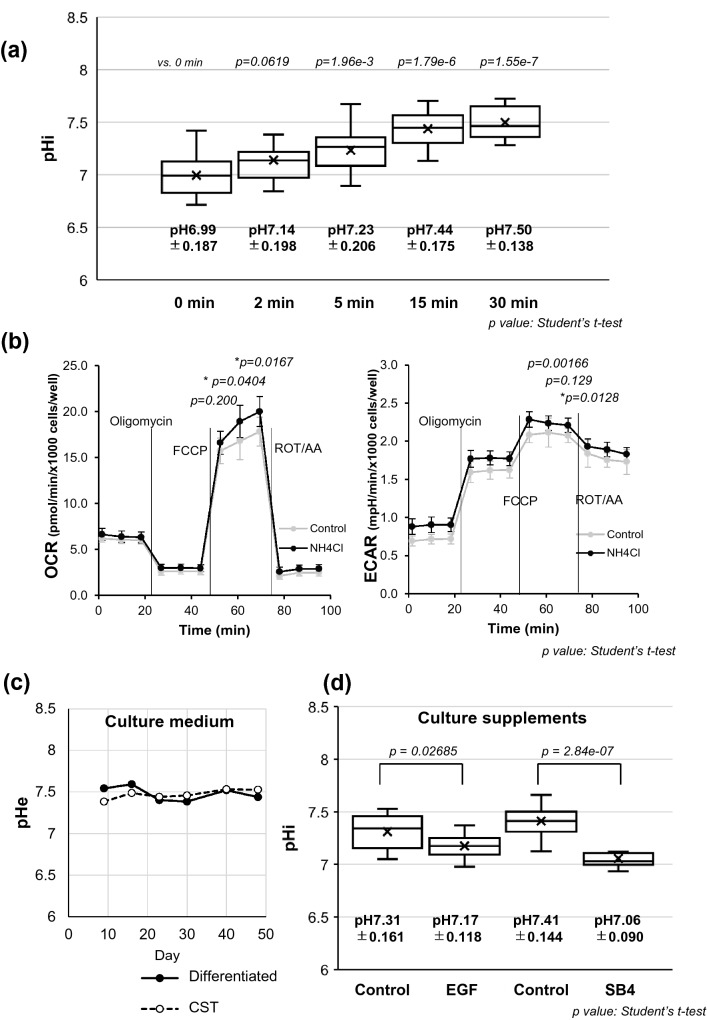
pHi regulation by external environmental factors. (**a**) The change in pHi with 20-mM ammonium chloride (NH_4_Cl). The differentiated SPs were seeded on 96-well plates and incubated for 6 days to become confluent. 0.4 µL of 5-M NH_4_Cl (final concentration: 20 mM) was added to each well, and the pHi was measured. (×) marks indicate the average of pHi (n = 6). (**b**) Real-time metabolic analysis with 20-mM NH_4_Cl treatment. The differentiated SPs were seeded on an XF24 flux analyzer plate and incubated overnight at 37 °C in 5% CO_2_. 2-mL PBS with 8 µL of 5 M NH_4_Cl (final concentration: 20 mM) or PBS only (control) was replaced from the culture medium in the XF24 flux analyzer plate and incubated at 37 °C in 5% CO_2_ for 30 min, and then replaced with minimal XF Dulbecco’s modified Eagle’s medium. (**c**) Extracellular pH (pHe) variation between differentiated and CST- SPs. Both SPs were obtained from the same donor. The pH of the culture medium was measured using a pH meter after collecting the culture medium and maintaining it at 37 °C in 5% CO_2_ for 2 h. (**d**) pHi changes with or without culture supplements (EGF or SB4) in differentiated SPs. (×) marks indicate the average of pHi (n = 6). The significance of the difference was assessed by Student’s *t*-tests after confirmation by F tests. *P* < 0.05 was considered statistically significant.

Next, we attempted to alter pHi non-physiologically by NH_4_Cl to gain preliminary insight into the effect of pHi on mitochondrial respiration. As expected from and consistent with previous publications^20, 29, 32, 34^, pHi increased in a time-dependent manner upon the addition of 20-mM NH_4_Cl (Fig. 4a). The shift of pHi to the alkaline side reduced the mitochondrial OXPHOS indexed by oxygen consumption rate (OCR) and increased extracellular acidification rate (ECAR) (Fig. 4b). To investigate the possible effect of extracellular pH (pHe) in the culture medium on pHi, pHe was monitored and we found that it remained almost constant, and no variation was found between the values for differentiated and CST SPs (Fig. 4c). Given our previous findings that culture supplements, such as epidermal growth factor (EGF) and SB431543 (SB4), originally thought to block the negative effect of transforming growth factor-β, can change mitochondrial OXPHOS^10^, we investigated whether these supplements would affect pHi. Differentiated CECs exposed to either EGF or SB4 for 24 h significantly decreased pHi (7.31 to 7.17 by EGF and 7.41 to 7.06 by SB4) (Fig. 4d). Although the extracellular pH was maintained by the buffering effect, culture supplements might participate in the pHi regulation, which might indirectly interfere with mitochondrial OXPHOS^10^.

### Ion transporter expression and pHi changes by inhibitor treatment

**Figure 5:**
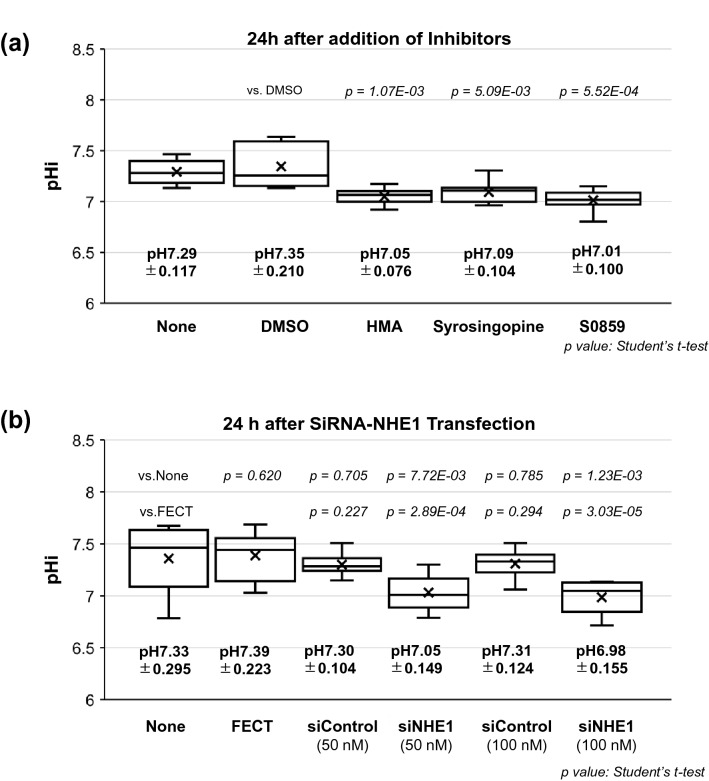
Ion transporter expression and pHi modulated by the transporter inhibitors. (**a**) pHi changes following an inhibitor treatment. 10-µM HMA (NHE1 inhibitor), S0859 (NBCe1 inhibitor), syrosingopine (MCT1 and MCT4 inhibitor), or DMSO (1:500 dilution) were administered in 6-well-plate differentiated SPs 24 h before the pHi measurement. (**b**) pHi changes caused by siRNA of the NHE1 treatment. The differentiated SPs were transfected with Silencer® Select siRNA of NHE1 (5 nM) or Silencer® Select Negative Control #1 siRNA using Dharma FECT Transfection Reagents according to the manufacturer’s instructions and incubated for 24 h. ( ×) marks indicate the average of pHi (n = 6). The significance of the difference was assessed by Student’s *t*-tests after confirmation by F tests. *P* < 0.05 was considered statistically significant.

Next, we tried to confirm whether the cell-intrinsic factors in CECs, such as ion transporters in Fig. 2, would certainly participate in regulating pHi. Previous reports have suggested that NHE1 is deeply involved in pHi regulation^19, 26, 35, 36^. Considering that the expression of NHE1 is positive only in differentiated SPs but almost null in CST SPs, we tried to explore the role of NHE1 in regulating pHi of differentiated SPs. The addition of HMA, S0859, or syrosingopine, the inhibitors of NHE1, NBCe1, and MCT4, respectively, dissolved in DMSO, reduced pHi in differentiated SPs (Fig. 5a). These results indicate that pHi could be changed by inhibiting the activity of ion transporters. Furthermore, to confirm the results, the effect of NHE1 siRNA (siNHE1) transduction on pHi in differentiated SPs was investigated. Treatment with siNHE1 resulted in decreased pHi, comparable to the above mentioned inhibitors (Fig. 5b).

### Inhibition of ion transporters elicited non-uniform modulation on mitochondrial respiration in CECs

**Figure 6:**
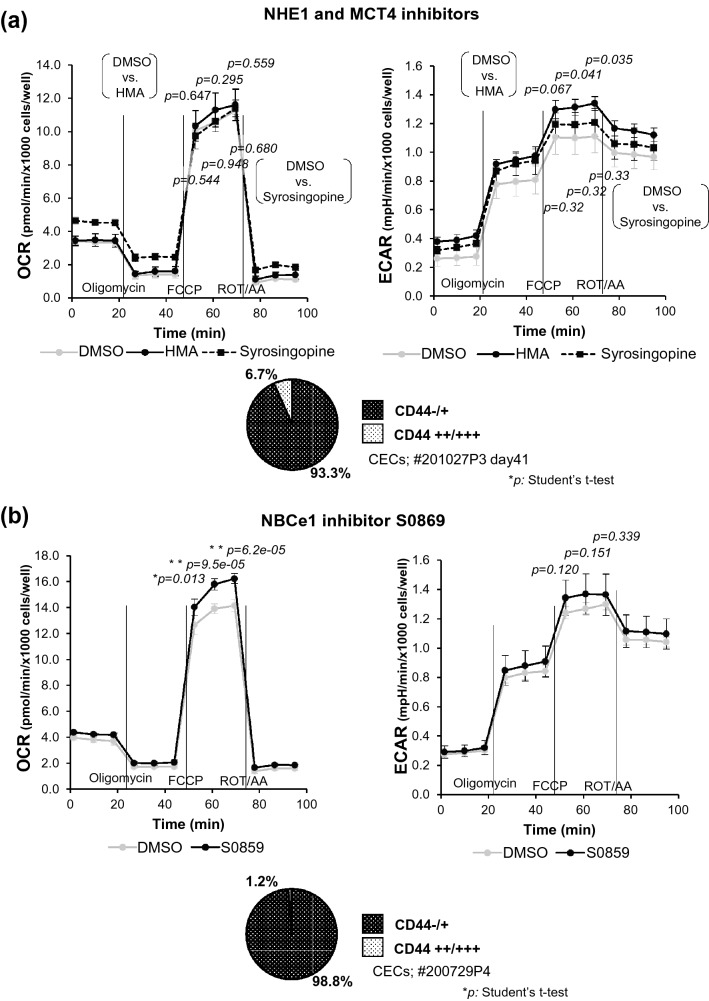
Modulation of mitochondrial respiration in CECs by the inhibition of ion transporters. The differentiated SPs were seeded onto the XF24 flux analyzer plates two days before the experiment and incubated overnight; then, each well was treated with the inhibitors (10-µM HMA, syrosingopine, S0859, or 1:500-diluted DMSO). The flux analysis was performed 24 h after the addition. The experiments were repeated minimally twice. (**a**) OCR/ECAR alteration in HMA and syrosingopine treatment. The spare respiratory capacity (pmol/min/ × 1000 cells/well) of HMA added CECs was 8.17 ± 0.68 vs 7.93 ± 0.15 of DMSO control (p = 0.582), whereas 6.89 ± 0.59 for syrosingopine added (p = 0.041) (**b**) OCR/ECAR alteration on S0859 treatment. The spare respiratory capacity for S0859 added CECs was 12.05 ± 0.31 vs 10.45 ± 0.28 of DMSO (p = 0.050). N = 3 for (**a**) and 5 for (**b**). The significance of the difference was assessed by Student’s *t*-tests after confirmation by F tests. *P* < 0.05 was considered statistically significant.

Finally, we investigated whether ion transporter inhibitors HMA, S0859, syrosingopine, and NHE1 siRNA would modulate mitochondrial respiration in differentiated CEC SPs. Note that 10-µM HMA or syrosingopine did not reduce OCR statistically significantly, whereas both of them increased ECAR statistically significantly (Fig. 6a). Of note, 10-µM S0859, a bicarbonate transporter NBCe1 inhibitor, increased both OCR and ECAR, indicating the elevation of the glycolytic flux and OXPHOS (Fig. 6b). The spare respiratory capacity (pmol/min/ × 1000 cells/well) of HMA added CECs was 8.17 ± 0.15 vs 7.93 ± 0.68 of DMSO control (*p* = 0.582), whereas 6.89 ± 0.59 for syrosingopine added CECs (*p* = 0.041). Spare respiratory capacity of S0859 added CECs was 12.05 ± 0.31 vs 10.45 ± 0.28 of DMSO control (*p* = 0.050) in the separate experiment.

## Discussion

Recent ground-breaking studies have shown that CECs are heterogeneous in their cell surface markers and metabolic phenotypes^7–10, 12^. The homogeneity of cultured cells with a standardized quality is a vital component to suffice the required governmental approval for a legal pharmaceutical application as a safe, effective, and stable cell-based therapy. The scientific rationalization of the superior clinical effect observed in a specified CEC, enriched and almost homogeneous with differentiated SPs, is indispensable for approval. The statement of only the stable expression of Na^+^/K^+^ ATPase and ZO-1 indicating barrier integrity does not fulfill the need to explain the finding that this specified CEC SPs elicited a higher clinical efficacy.

One of the principal aims of this study was to provide a cogent insight into the discrepancy in clinical efficacy between differentiated and CST-CEC SPs. In a series of previous studies, we succeeded in discriminating these SPs by their metabolic signatures^9, 10^. In the current study, we first found functional phenotypes to discriminate these SPs, namely, the selective expression of AQP1 and SLC4A11, as well as ZO-1 (Figs. 2). AQP1 is, reportedly, located at the apical side of HCEs^13^. The AQPs are integral membrane proteins to transport water across cell membranes in response to osmotic gradients^37^. Casey and his associates newly identified SLC4A11, a member of bicarbonate transporters, as a water-channeling protein at the basolateral side of HCEs^13^. The polarized expression of AQP1 and SLC4A11 only in fully differentiated CEC SPs may be critically instrumental to explain the clinical effect in these CEC SPs^5, 6^. The well-oriented localization of ZO-1 in the tight junction (Fig. 2) also supported this interpretation by the findings of Fischbarg et al. that trans-endothelial fluid movements can be triggered as long as there is tight junction integrity^38^. Consequently, CEC SPs expressing AQP1, SLC4A11, and ZO-1 may cooperatively mediate an efficient trans-endothelial water efflux from the stromal side to the anterior chamber. Considering the role played by oxidative stress in the pathogenesis of several corneal diseases^39–41^, SLC4A11 may play an additional role in regulating this oxidative stress^42^. One more critical issue of this study was to account for the role of pHi, distinct between differentiated and CST SPs. To the best of our knowledge, there have been no published reports on the linkage of the metabolic rewiring of CECs to pHi homeostasis. Intracellular pH (pHi) is stringently controlled as a component of numerous basic cellular systems^26^. While NHE proteins are ubiquitously expressed to regulate pHi, other pHi regulators become critical only under specific conditions^32, 43^. Elevated pHi may promote cell growth through the intrinsic pH sensitivity of metabolic enzymes, such as phosphofructokinase and lactate dehydrogenase (LDH). Consistent with this notion, pHi was elevated in proliferative CST SPs (Fig. 3b), which showed the elevation of LDH in our previous study^9^. Among CEC SPs, proliferative CST SPs switched to a glycolytic metabotype, resulting in increased lactate secretion^9^. In contrast, differentiated SPs, dominant in OXPHOS, may theoretically cause a higher lactate gradient from the basolateral to the apical side than would CST SPs in reconstituted HCE tissues. Bonanno and associates reported that the passive fluid influx is offset by an outward active “pump,” thereby maintaining corneal hydration and transparency^44, 45^. They described that the endothelium can form the basolateral to apical [lactate] gradients needed for the osmotic water flux, and the reduced lactate efflux leads to the increased corneal [lactate] and corneal edema^33^. The observed selective expression of MCT4, which functions to efflux monocarboxylic acid and lactic acid, in differentiated SPs may further affect the superlactate of the [lactate] gradients. Fully differentiated CEC SPs exhibited a lower pHi of around 7.0–7.2, compared with the proliferative CST SPs with an alkaline pHi > 7.5 (Fig. 3b). The latter SPs led to an elevated glycolysis flux, in addition to OXPHOS, rather than differentiated SPs with decreased lactate production^9^.

Intriguingly, the pHi alteration followed almost the same culture kinetics, both with the reported mitochondrial respiration^10^ and the reduced CD44 expression until the mitochondrial reserve was stabilized in approximately five weeks after the cell seeding^10^ (Fig. 3a, b). However, while all the inhibitors, HMA, S0859, syrosingopine, and NHE1 siRNA, acidified the pHi of differentiated CEC SPs in accordance with their thus far known functions (Figs. 5a and b), the influences on mitochondrial respiration were not uniform at first glance (Fig. 6). Differentiated CECs exposed to either EGF or SB4 significantly decreased pHi (Fig. 4d), and this finding was fully consistent with our previous findings that these agents reduce the mitochondrial OXPHOS^10^ and that the culture in the presence of either EGF or SB4 reduces the ratio of differentiated CEC SPs in the produced CECs^8^. A further precise and extensive investigation is required to clarify the association of pHi with the mitochondrial functions in CECs, which is far beyond the aim of this study.

Na^+^/K^+^-ATPase, NHE1, NBCe1, and MCT4 were elevated in fully differentiated SPs (Fig. 2). This finding will help further define CEC SPs efficacy in clinical settings.

Finally, note that pHi-modulated mitochondrial functions are crucial in deciding the cell’s fate. In most of the cell lines, NHE1 is more highly expressed in proliferative or transformed or cancer cells^20, 29, 46^ according to the principle that an increased pHi and increased glycolysis flux are two sides of the same coin^32^. Depending on the culture stages, the expression of ion transporters will be plastic and fragile. The expression of CD44 was downregulated in NHE1 expressing differentiated SPs with neutral pHi (Figs. 2 and 3b). The differentiation was supposed to be caused by the supplemented Rock inhibitor Y27632^8^. CD44, well known to be repressed by wild-type p53^47^, promotes glycolysis, which is consistent with our previous finding of upregulated p53 in CD44-/+ differentiated SPs^48^. CD44 bound to hyaluronic acid activates Rock (Rho kinase) to mediate NHE1 phosphorylation, leading to the induction of cell transformation. The inhibition of ROCK or NHE1 by a ROCK inhibitor, Y27632, or a NHE1 inhibitor reduces intracellular acidification and prevents oncogenic transformation^49^. Note that either decreased or increased pHi contributes to stem cell differentiation depending on the cell type: human mesenchymal stem cells (MSCs)^22^ or mouse embryonic stem cells^20^. In addition, Fliegel et al.^31^ reported a novel link between NHE1 and the regulation of EMT.

**Figure 7:**
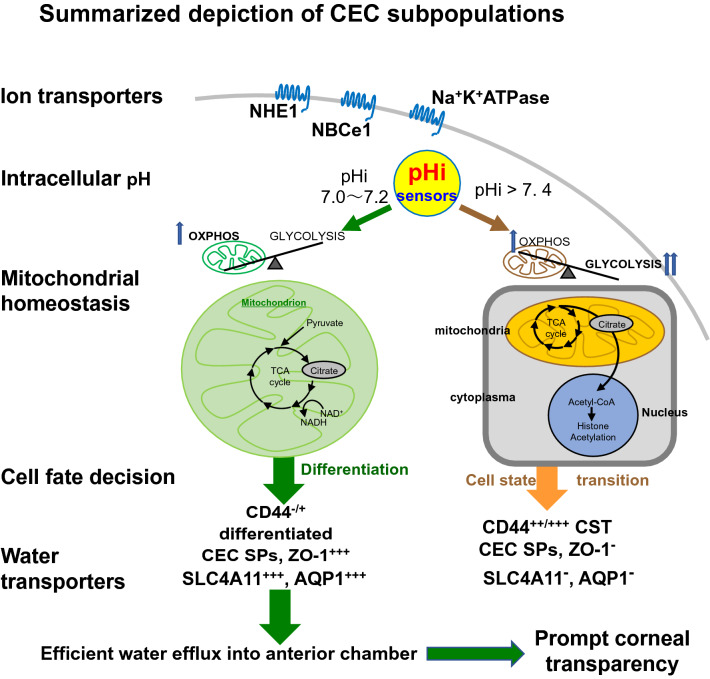
Depiction of CEC subpopulations. The summarized depiction of the phenotypic and functional characteristics of two differentiated versus CST-CEC SPs presented in a current study, together with our previously reported findings^5–7, 9, 10, 12^. “We attribute the differences in intracellular pH between the two SPs to variations in the expression profile of specific ion transporters and mitochondrial functions; in differentiated CECs, ion transporters such as NBCe1 and NHE1 contribute to maintain neutral intracellular pH. The differentiated SPs also express AQP-1 and SLC4A11 working for the water efflux. However, the CST-SPs, deficient in the expression of these ion transporters, AQP-1 and SLC4A11 and not well working in the water efflux, are non-efficacious endothelial cells in cell injection therapy. The integrity of barrier function here verified by the expression of ZO-1, expressed only in differentiated SPs, co-operatively functions in generating osmotic gradients, thereby resulting in an efficient fluid/water efflux from the stroma to the anterior chamber. Refer to the detailed discussion in the text.”

In conclusion, we hypothesized that in differentiated CECs, mitochondrial homeostasis maintained in neutral pHi might cooperate with the ion transporters in generating osmotic gradients, thereby resulting in an efficient water efflux from the stroma to the anterior chamber. This scheme might also accelerate the differentiation into CD44-/+  CEC SPs. This implies the need to regenerate HCE by the injection of highly purified with differentiated CEC SPs in clinical settings. The summarized hypothetical cell fate decision; the alteration of the phenotypic and functional characteristics of differentiated- and CST- CEC SPs is shown in Fig. 7, together with our previous findings.

Given a range of molecular mechanisms thus far proposed to explain FECD and CHED pathology, further extensive studies on the role of ion transporters and water transporters in the in vitro human CEC culture model to simulate in vivo HCEC functional failures might open a new avenue for the diagnosis of these dysfunctions beyond cell injection therapy.

## Materials and methods

### Human corneal endothelial cell donors

The human tissue used in this study was handled in accordance with the tenets set forth in the Declaration of Helsinki. HCECs were obtained from human donor corneas supplied by CorneaGen (Seattle, WA, USA) Eye Bank and then cultured prior to the experimental analysis. All the procedures followed the acquisition of the tissues described previously^7–12^, including the informed written consent for eye donation for research.

All procedures were conducted in accordance with the ARVO Statement for the Use of Human Materials in Ophthalmic and Vision Research, and the all the details of the current experimental study protocols were approved by the Institutional Ethical Committee of Kyoto Prefectural University of Medicine, Kyoto, Japan.

### Reagents

Rho-associated protein kinase (ROCK) inhibitor Y-27632 and epidermal growth factor (EGF) were obtained from Wako Pure Chemical Industries, Ltd. (Osaka, Japan), and a p38 MAP kinase inhibitor SB203580 was obtained from Cayman Chemical (Ann Arbor, MI, USA). SB431543 (SB4), originally thought to block the negative effect of transforming growth factor-β on cultures, Dulbecco’s modified Eagle’s medium, high glucose (DMEM-HG), and fetal bovine serum were obtained from Thermo Fisher Scientific (Waltham, MA, USA), and plastic culture plates were obtained from Corning (Corning, Inc., Corning, NY, USA). Ammonium chloride (NH_4_Cl), an inhibitor of NHE1; 5-(N,N-hexamethylene) amiloride (HMA), an inhibitor of NBCe1; S0859, an inhibitor of monocarboxylate transporters 1 and 4 (MCT1, 4); and syrosingopine were obtained from Sigma-Aldrich, Inc. (St. Louis, MO, USA). Unless otherwise stated, all other chemicals were obtained from Sigma-Aldrich, Inc. (St. Louis, MO, USA).

### Cell cultures of HCECs

HCECs were cultured according to the previously published protocols, but with modifications^7–9, 11^. Differentiated and CST CEC SPs were established according to the procedures described previously^7–9, 11^. The former SPs elicited the proportion of CD44-/+ to be more than 95%, while the latter was designated as the SPs eliciting the proportion of less than 70%. Flow cytometry analyses of the CECs were described previously. The CECs were collected and suspended at 4 × 10^6^ cells/mL in the flow cytometry (FACS) buffer described^7^. The antibody (Ab) solutions were all the same as described^7^.

### Measurement of culture medium, pH

The pH of the culture medium was measured using a pH meter (F-72S, HORIBA, Ltd., Kyoto, Japan) after collecting the culture medium and maintaining it at 37 °C in full humidity with 5% CO_2_ for 2 h.

### Measurement of pHi in CECs

The pHi of CEC SPs was measured by using the cell-permeable probe 3′-O-acetyl-2′,7′-bis(carboxyethyl)-5,6-carboxyfluoresceinacetoxymethylester (BCECF-AM special packaging; Dojindo Laboratories. Kumamoto, Japan). Cells were detached with TrypLE Select 10 × (Thermo Fisher Scientific) for 15 min at 37 °C, washed twice with the HEPES buffer (152-mM NaCl, 5-mM KCl, 5-mM glucose, 20-mM HEPES), and then incubated with BCECF-AM for 30 min at 37 °C in full humidity with 5% CO_2_. Cells were washed twice with the HEPES buffer, and 150 µL/well of the cell suspension solution was poured into a 96-well plate. The fluorescence intensity was determined using a fluorescent plate reader with an excitation wavelength of 488 nm (GloMax Explorer; Promega Corporation, Madison, WI, USA). In this study, we used Dojindo’s assay kit (Dojindo Laboratories). The procedures for calibrating the BCECF fluorescence were described previously^12^.

### pHi regulation by ammonium chloride

First, the change in pHi with ammonium chloride, NH_4_Cl, was measured. Cells were seeded on 96-well plates (40,000 cells per well) and incubated for 6 days to become confluent. The culture medium was changed once on day 3. All the medium was then removed, and the culture was washed twice with 100 µL of the HEPES buffer. Next, 35 µL of 1-mM BCECF-AM (final, 5 µM) was added to 7 mL of the 1:1 mixture of the HEPES buffer and the Nancy medium (FBS). Next, 100 μL of this mixture was administered to each well, and the sample was incubated at 37 °C in full humidity with 5% CO_2_ for 30 min. The cells were then washed twice with 100 µL of the HEPES buffer, and then 100 μL of the HEPES buffer was administered to the samples. For the calibration, 3 µL of 2-mg/mL Nigericin/EtOH was added to 600 µL of each pH calibration buffer (final, 5 µg/mL), and 100 µL of this solution was added. Next, 0.4 µL of 5-M NH_4_Cl (final, 20 mM) was added, and the pHi was measured. For real-time metabolic analysis with NH_4_Cl treatment, PBS with 8 µL of 5-M NH_4_Cl (final, 20 mM) or PBS only (control) was replaced from the culture medium in the XF24 flux analyzer plate and incubated at 37 °C in full humidity with 5% CO_2_ for 30 min before the cell culture replacement with minimal XF Dulbecco’s modified Eagle’s medium.

### Quantitative real-time polymerase chain reaction (qRT-PCR)

Total RNA was extracted from the CECs with the miRNeasy mini kit (QIAGEN, Hilden, Germany). The cDNA was synthesized using a high-capacity cDNA reverse transcription kit with an RNase inhibitor (Thermo Fisher Scientific). A polymerase chain reaction was performed using TaqMan Fast Advanced Master Mix (Thermo Fisher Scientific) and TaqMan Gene Expression Assays (inventoried) (Thermo Fisher Scientific) under the conditions described earlier^7, 10^. The levels of *Na*^+^*/K*^+^ *ATPase (ATP1A1), AQP 1, SLC4A11, NBCe1, NHE1, MCT1, and MCT4* were normalized to the level of *GAPDH.* The results were presented as 2^−ΔCt^ (relative units of expression).

### Immunofluorescence analysis of ion transporters

The cells were fixed with ice-cold methanol for 10 min and then permeabilized with PBS (-) containing 0.1% Triton X-100 at room temperature (RT) for 15 min. After blocking nonspecific reactivity with 1% BSA in PBS(-) at RT for 1 h, the cells were incubated at 4 °C overnight with antibodies against Na^+^/K^+^-ATPase (No. 05-369, Merck), ZO-1 (No. 33-9100 and No. 61-7300, Thermo Fisher Scientific), AQP1 (ab168387, Abcam), SLC4A11 (HPA018120, Merck), NBCe1 (sc-515543, Santa Cruz Biotechnology Inc.), NHE1 (ab67314, Abcam), MCT1 (NBP1-59656, Novus Biologicals), and MCT4 (ab234728, Abcam). After washing with PBS (-), the cells were incubated for 1 h with fluorescent Alexa-Fluor secondary antibodies. The cell nuclei were stained with 40,6-diamidino-2-phenylindole (DAPI) (No. 340-07971, Dojindo, Laboratories). The signal was detected using a fluorescence microscope (BZ-9000; Keyence Corp., Osaka, Japan).

### Mitochondrial respiration assay

A real-time metabolic analysis of live CECs was performed using the Seahorse XFe24 extracellular flux analyzer (Agilent Technologies, Santa Clara, CA, USA). CECs cultured were seeded on an XF24 flux analyzer plate. For the Mito Stress test were performed according to the manufacturer’s protocol. For Mito Stress, cell culture medium or Optisol GS was replaced 1 h before the assay with minimal XF DMEM medium supplemented with 2 mmol/L glutamine, 10 mmol/L glucose, and 1 mmol/L sodium pyruvate (pH 7.4). Oxygen consumption rate (OCR) and extracellular acidification rate (ECAR) was analyzed at basal conditions and after sequential injections of 1 μM oligomycin, 1 μM FCCP, 0.5 μM rotenone and antimycin A. The assay results were normalized on the basis of viable cell number counted by Cell Insight NXT (Thermo Fisher Scientific).

### Treatment with HMA, S0859, and syrosingopine

The pHi and mitochondrial respiration 24 h after the addition of HMA, S0859, and syrosingopine were explored after preliminary tests of the optimal time and concentration to achieve a significant change in pHi. As there are no reports on the use of HMA, S0859, and syrosingopine on CECs, we first confirmed the absence of non-specific cell death and applied these inhibitors at a final concentration of 10 μM, except otherwise stated. HMA, S0859, and syrosingopine were dissolved in DMSO at 5 mM and administered at a concentration of 1:500 in a 6-well plate or a 96-well plate (final concentration: 10 µM). The measurements (biological repetition, *n* > 5) were performed 24 h after the addition of these inhibitors. For the flux analysis after the inhibitor treatment, the cells were seeded onto XF24 flux analyzer plates (40,000 cells per well) two days before the experiment and incubated overnight; then, each well was treated with the inhibitors. The flux analysis was performed 24 h after the addition. The experiments were repeated at least twice.

### RNA interference

The cells were transfected with Silencer® Select siRNA of NHE1 (5 nM) or NBCe1 (5 nM) or Silencer® Select Negative Control #1 siRNA (Thermo Fisher Scientific) using DharmaFECT Transfection Reagents (Horizon, Cambridge, UK) according to the manufacturer’s instructions, and incubated for 24 h. The sequences of siRNAs used were NHE1 siRNA, 5′-CGAAGAGAUCCACACACAGtt-3′, and 5′-CUGUGUGUGGAUCUCUUCGtt-3′; and NBCe1 siRNA, 5′-AAAGAAGGAGGAUGAGAAGtt-3′, and 5′-CUUCUCAUCCUCCUUCUUUtt-3′.

### Statistical analysis

The data were analyzed using EZR (Saitama Medical Center, Jichi Medical University, Saitama, Japan), which is a graphical user interface for R (The R Foundation for Statistical Computing, Vienna, Austria)^50^. More precisely, it is a modified version of the R commander designed to add statistical functions frequently used in biostatistics. An unpaired two-tailed Student’s *t*-test or Mann–Whitney U test was performed when applicable. The data were considered statistically significant at a *P* value of less than 0.05.

## Supplementary Information

Supplementary Information.
