# Supplementary material for: Intracellular pH affects mitochondrial homeostasis in cultured human corneal endothelial cells prepared for cell injection therapy
Source: Sci Rep. 2022 Apr 15;12:6263. doi: 10.1038/s41598-022-10176-1 (PMC9012833; doi:10.1038/s41598-022-10176-1)
Supplement: Supplementary file 1 — Supplementary Information. [file 41598_2022_10176_MOESM1_ESM.docx]

**Intracellular pH affects Mitochondrial Homeostasis in Cultured Human Corneal Endothelial Cells prepared for Cell Injection Therapy**

Hideto Deguchi,^1^ Tomoko Yamashita,^1^ Nao Hiramoto,^1^ Yohei Otsuki,^1^ Atsushi Mukai,^1^ Morio Ueno,^1^ Chie Sotozono,^1^ Shigeru Kinoshita^2^ and Junji Hamuro^1,*^

^1^Department of Ophthalmology, Kyoto Prefectural University of Medicine, Kyoto, Japan

^2^Department of Frontier Medical Science and Technology for Ophthalmology, Kyoto Prefectural University of Medicine, Kyoto, Japan

Supplementary Figure

The mitochondrial respiration activity plot profile. Vertical dashed lines indicate assay drug injections. Colored boxes indicate the proportion of the overall profile corresponding to basal respiration, ATP produced, proton leak, maximal respiration, spare respiratory capacity, and non-mitochondrial respiration.

OCR; oxygenconsumption rate

O: Oligomycin,

F: Carbonyl cyanide-p-trifluoromethoxyphenylhydrazone,

R/A: Rotenon/Antimycin A

Adapted from

Aldrich BT, Schlötzer-Schrehardt U, Skeie JM, Burckart KA, Schmidt GA, Reed

CR, Zimmerman MB, Kruse FE, Greiner MA. Mitochondrial and Morphologic

Alterations in Native Human Corneal Endothelial Cells Associated With Diabetes

Mellitus. Invest Ophthalmol Vis Sci. 2017;58:2130-2138.
